# Changes in Acute Phase Proteins in Bitches after Laparoscopic, Midline, and Flank Ovariectomy Using the Same Method for Hemostasis

**DOI:** 10.3390/ani10122223

**Published:** 2020-11-27

**Authors:** Ayla Del Romero, Belén Cuervo, Pau Peláez, Laura Miguel, Marta Torres, Marc Yeste, Maria Montserrat Rivera del Alamo, Camila P. Rubio, Mónica Rubio

**Affiliations:** 1 Bioregenerative Medicine and Applied Surgery Research Group, Department of Animal Medicine and Surgery, University CEU—Cardenal Herrera, CEU Universities, C/Tirant lo Blanc, 7, Alfara del Patriarca, 46115 Valencia, Spain; ayla.delromero@uchceu.es (A.D.R.); pau.pelaez@uchceu.es (P.P.); laura.miguel@uchceu.es (L.M.); marta.torrestorrillas@uchceu.es (M.T.); mrubio@uchceu.es (M.R.); 2 García Cugat Foundation CEU-UCH Chair of Medicine and Regenerative Surgery, University CEU-Cardenal Herrera, CEU Universities, C/Tirant lo Blanc, 7, Alfara del Patriarca, 46115 Valencia, Spain; 3 Biotechnology of Animal and Human Reproduction (TechnoSperm), Institute of Food and Agricultural Technology, University of Girona, E-17003 Girona, Spain; marc.yeste@udg.edu; 4 Unit of Cell Biology, Department of Biology, Faculty of Sciences, University of Girona, E-17003 Girona, Spain; 5 Unit of Animal Reproduction, Department of Animal Medicine and Surgery, School of Veterinary Medicine, Autonomous University of Barcelona, E-08193 Bellaterra (Cerdanyola Del Vallès), Spain; mariamontserrat.Rivera@uab.cat; 6 Interdisciplinary Laboratory of Clinical Analysis (Interlab-UMU), Veterinary School, Campus of Excellence Mare Nostrum, University of Murcia, Espinardo, 30100 Murcia, Spain; camila.peres@um.es

**Keywords:** acute phase proteins, surgical trauma, ovariectomy, laparoscopy

## Abstract

**Simple Summary:**

Ovariectomy is a common surgical procedure in veterinary medicine, with many techniques involved. The aim of this study is to objectively evaluate the acute phase response by measuring the changes in a panel of acute phase proteins after applying three different ovariectomy techniques. C-reactive protein values showed increases of lower magnitude after laparoscopic ovariectomy compared with other techniques, indicating that this surgical technique induces a reduced inflammatory response and tissue damage. The use of this procedure is in agreement with the current tendency to use minimally invasive procedures for ovariectomy.

**Abstract:**

Acute phase proteins (APP) are biomarkers of systemic inflammation, which allow monitoring the evolution of diseases, the response to treatments, and post-operative complications. Ovariectomy (OVE) is frequently performed in veterinary medicine and can be a useful model to evaluate surgical trauma and inflammation in the bitch. The objective was to investigate and compare the acute phase response (APR) after applying three different OVE techniques by measuring serum levels of C-reactive protein (CRP), haptoglobin (Hp), albumin (Alb), and paraoxonase-1 (PON-1). Forty-five intact bitches were included in the study, being randomly distributed into three groups: laparoscopic OVE (L), midline OVE (M), and flank OVE (F). Serum CRP, Hp, Alb, and PON-1 were measured before surgery, 1, 24, 72, and 168 h post-intervention. CRP levels increased significantly 24 h post-surgery in the M and F groups, but no significant variation was observed in the L group at any time of the study period. Hp was significantly higher in group L than in group F 72 h post-surgery. Alb and PON-1 showed no statistical difference among groups or among sampling periods. CRP response suggests that the use of laparoscopic procedures produce lower inflammation compared to open conventional approaches when performing OVE in the bitch.

## 1. Introduction

Surgical procedures in veterinary medicine are aimed to help the animal recover its health status or promote the prevention of diseases. Any surgical procedure implies a surgical stress that will induce a response composed of a set of metabolic, hormonal, and inflammatory reactions, which allow the body to adapt to trauma and recover damaged tissues [1,2]. These inflammatory reactions are part of the acute phase response (APR). APR consists of a systemic reaction of the organism to local or systemic disorders in its homeostasis, caused not only by surgical trauma, but also by infection, tissue injury, neoplastic growth, or immunological disorders [3,4,5]. During the APR, changes in the synthesis and release of acute phase proteins (APP) induced by pro-inflammatory cytokines occur. These proteins are classified into positive APP (if serum concentration increases), or negative APP (if serum concentration decreases) [5,6].

C-reactive protein (CRP) is a major positive APP that has been studied in several inflammatory processes and it is used to monitor the response to treatment in dogs [5,7,8]. CRP can increase up to 95 times after surgery, and this increase is related to the degree of tissue injury in dogs. Thus, when different surgical procedures are compared, CRP values correlate with the tissue trauma [9,10,11]. Changes in the post-operative period report maximum concentrations of CRP after approximately 12–24 h [12] and significantly increased concentrations have been demonstrated for several days after surgery, varying from 7 days [13] to 17 days [14].

Haptoglobin (Hp) is a moderate positive APP involved in host defense responses to infection and inflammation [5]. Increases in Hp concentration have been described after surgical trauma in dogs, as well as other species, such as cats [15,16,17]. Changes in Hp can be detected at 24 h after a surgical procedure, showing the peak concentration 3–4 days after surgery [5].

Albumin (Alb) is the most abundant protein in the blood and is considered a negative APP [18]. Low Alb is related to poor outcomes in human medicine and it has been used to monitor the effects of different treatments in leishmania in dogs [19].

Paraoxonase-1 (PON-1) is an enzyme associated with high-density lipoproteins (HDL) and considered a negative APP [20]. There are few studies on this inflammatory biomarker in dogs in different medical situations [2,21,22], but its changes after surgery are less described [23].

Surgical neutering is commonly performed in the veterinary practice, as surgical sterilization has several proven benefits, including population control, prevention of some reproductive tract disorders, and elimination of undesirable behaviors associated with hormonal cycling [24,25]. Described techniques for bitch surgical sterilization are ovariectomy (OVE) and ovariohysterectomy (OVH), being both equally effective. OVE has potential advantages as smaller incisions, better viewing of the ovarian vessels and lower risk of surgical complications [26,27,28,29,30]. However, this technique should not be used if there is uterine disease or to prevent uterine neoplasm [26]. The surgical approach to OVE in the bitch has been debated over the last years [31,32,33,34,35]. Midline and flank laparotomies, known as open or conventional OVE, have been the most frequent approaches while laparoscopic OVE has recently gained interest in veterinary practice [36,37]. Many advantages are described for laparoscopic procedures over conventional laparotomies in both human and veterinary medicine: decreased post-operative stress and pain, shorter recovery periods, decreased hospitalization stay, better surgical wound outcome, and improved visualization of the abdominal cavity [38,39,40,41,42,43,44]. However, most of these parameters might be affected not only by the intensity of the surgical trauma, but also by some other factors, such as age, health status, breed, or character of the animal [45]. Laparoscopic procedures also have some disadvantages over open conventional procedures: high cost of the equipment, the need of an assistant, higher learning curve, longer surgical time, and the requirement of intra-abdominal carbon-dioxide (CO_2_) insufflation (pneumoperitoneum) [43,46,47,48]. The latter has been associated with several complications, which include visceral pain, local peritonitis, hemodynamic changes, oxidative stress, and inflammation [49,50,51,52,53].

In human medicine, APR after surgical trauma is well described, being this response proportional to the level of tissue injury [54,55,56,57]. In veterinary medicine, changes in APP in the post-operative period have also been described, with CRP being one of the most studied biomarkers [10,11,17,58,59]. These inflammatory biomarkers have also been studied in conjunction with the leucogram to monitor the post-operative period in dogs [60,61], as well as after surgical trauma in other species such as cows [62,63]. Specific studies on APR after surgical neutering (OVE or OVH techniques) are described in both dogs and cats [11,16,28,58].

Comparison among several types of surgery of different magnitude shows that APR increases according to the degree of the surgical trauma [10]. Changes in the APP have also been studied in dogs undergoing minimally invasive surgery of different types, showing major increases in the APP than in the open techniques [42]. Some studies compare conventional midline laparotomy with mini-laparotomy techniques or laparoscopic-assisted techniques [15,64], but purely laparoscopic technique is less represented [10,16,65]. Different techniques for hemostasis of the ovarian pedicle (sutures, ties, sealing devices) are used among these studies, which may cause variations in the post-surgical inflammation [37,39,43,44,66]. To the authors’ knowledge, there is no published evidence of the APR in bitches after OVE, comparing midline, flank, and laparoscopic techniques using the same method for hemostasis of the ovarian and uterine pedicles.

Therefore, the objective of this study was to evaluate the effect of three different OVE techniques, namely laparoscopic, midline and flank OVE, on the kinetics of two positive APP (CRP and Hp) and two negative APP (Alb and PON-1), and to compare the effects of inflammation by the three procedures in the postoperative period. Knowing this information regarding inflammation could help to better select the technique for OVE in daily practice.

## 2. Materials and Methods

### 2.1. Animals

Forty-six entire healthy bitches of different breeds were initially included (Table 1). The dogs aged between 5 and 48 months old, and weighed between 2.5 and 51 kg. The study was approved by the Ethics Committee for Animal Experimentation of the University CEU-Cardenal Herrera (2018/VSC/PEA/0195). All animal owners signed a consent form after having been explained the relevant project information.

Before surgery, dogs underwent a thorough clinical examination to ensure that they were completely healthy. Complete blood cell count and biochemistry profile, thoracic radiographs and electrocardiogram were performed. Uranotest^®^ Quattro (Urano Vet SL, Barcelona, Spain) was used to detect antibodies for *Leishmania infantum*, *Ehrlichia canis and Anaplasma platys*, and to detect antigens for *Dirofilaria immitis.* Only dogs assigned to ASA (American Society of Anesthesiologists) category 1 (normal healthy animals) [67], and with negative results on the Uranotest^®^ were included. As the surgical technique consisted of OVE, any animal in which OVH was recommended for clinical reasons, was excluded. If post-operative complications appeared, the animal was also excluded from the study.

### 2.2. Study Groups

The forty-six bitches were randomly divided into three groups, depending on the surgical technique: 16 animals were located in group L (laparoscopic OVE), 15 animals in group M (midline OVE), and 15 animals in group F (flank OVE).

### 2.3. Anesthesia and Antibiotic Prophylaxis

Anesthetic protocol consisted in premedication with dexmedetomidine (5 µg/Kg, Dexdomitor^®^, Orion Corporation, Espoo, Finland) and methadone (0.3 mg/Kg, Semfortan^®^, Eurovet Animal Health BV, Bladel, Holland) intramuscular. Vital signs (respiratory rate, heart rate, blood pressure, and temperature) were taken 15 min after premedication and prior to induction with propofol (2–5 mg/Kg/dose effect, Propofol Lipuro^®^ 1%, Melsungen, Alemania) intravenously (IV).

Anesthesia was maintained with sevoflurane (SevoFlo^®^, Zoetis, Louvain-la-Neuve, Belgium) in 50% oxygen/air mixture. Intermittent positive pressure ventilation (VPPI) was applied to ensure normocapnia and the volume was regulated in order to keep the end-tidal CO_2_ at normal levels (35–45 mmHg). All animals received IV fluid therapy with Ringer-Lactate solution (5 mL/Kg/h, Braun^®^, Barcelona, Spain) during the surgical procedure, and a single dose of cefazolin (20 mg/Kg/IV, Cefazolina Normon^®^, Normon laboratories, Madrid, Spain) before starting the surgeries. Intraoperative monitoring consisted of body temperature, electrocardiogram, capnography, pulse oximetry, noninvasive blood pressure, and oxygen and vapor concentrations.

### 2.4. Surgical Procedures

Three surgical techniques for OVE were applied, laparoscopic (L), midline (M), and flank (F) approach. All techniques were performed by the same surgeon with the help of an assistant. The surgical area was clipped and scrubbed (abdomen for group L and M and flank for group F). In groups M and F, the distance from xiphoid to pubis was measured, to calculate the size of the incision (20% of this length) and standardize the surgical wound.

OVE technique was carried out depending on the group. For group M, an incision was made on the abdominal area, caudal to the umbilicus, to approach the linea alba after dissecting the subcutaneous tissue. For group F, a single dorsoventral incision in the skin was made on the left flank of the animal, starting caudal to the midpoint between the last rib and iliac crest. The subcutaneous tissue was dissected and the fibers from the muscle bellies of the external abdominal oblique, internal abdominal oblique and transverse abdominal were opened separately, according to their fiber direction as described in the literature [32]. In both groups, after identifying the ovaries, a vessel sealing device LigaSure™ (Medtronic [formerly Covidien], Minneapolis, MN, USA) was used for the sealing of the suspensory ligament, the ovarian artery and vein, and the uterine artery and vein, just caudally to the ovaries so the uterus was not excised.

For group L, a two cannulas laparoscopic technique [68] was used, so each animal had two incisions (1 cm cranial and 1 cm caudal to the umbilicus) that were the same size of the cannulas (5 mm). Pneumoperitoneum was stablished by insufflating CO_2_ to a pressure of 7 mm Hg. The patient was rotated into right lateral oblique recumbency for removing the left ovary and subsequently into left lateral oblique recumbency for removing the right ovary. A circular 0-size needle was used during each procedure to transabdominally stabilize the ovary against the abdominal wall. Ovaries were removed through the cranial 5-mm cannula after sealing the ovarian vessels using the LigaSure™ (Medtronic [formerly Covidien], Minneapolis, MN, USA) sealing device.

In group M, linea alba was closed using a simple continuous pattern, and in group F, muscles were sutured in two layers with the transverse and internal oblique muscle together, and the external oblique muscle as a second layer, as described in the literature [33,69]. Subcutaneous tissue was sutured with a simple continuous pattern, and skin was closed with an intradermic pattern. For all of the layers, absorbable monofilament glyconate suture was used (Monosyn^®^, B. Braun VetCare SA, Barcelona, Spain). For group L, the same type of suture was placed to close the abdominal wall at each cannula site, using a cruciate pattern. Subcutaneous tissue was closed using a cruciate pattern and surgical glue was used to seal the skin.

No complications related to anesthesia or intraoperative hemorrhage were observed during surgery in any case.

Post-operative care consisted of hospitalization during the first 24 h and analgesia receiving methadone (0.2 mg/kg/6 h Semfortan^®^, Eurovet Animal Health BV, Bladel, Holland) (IM). Only one dog experienced post-operative complications (a dog in group L that licked at the wound site), and it was excluded from the study

### 2.5. Blood Sampling

Blood samples (3 mL) were taken by jugular venipuncture just before ovariectomy (B) and at 1, 24, 72, and 168 h post-surgery. Blood was allowed to clot at room temperature for 30 min and centrifuged (3000 rpm, 10 min). The obtained serum was separated and kept in Eppendorf microtubes (Eppendorf, Hamburg, Germany) at −80 °C until analysis.

Serum concentration of CRP was measured using a human immunoturbidimetric test (CRP OSR 6147 Olympus Life and Material Science Europe GmbH, Lismeehan, O’Callaghan’s Mills, Co., Clare, Ireland). A colorimetric method (kit haptoglobin TrideltaPhase range SAA kit, Tridelta Development Ltd., Co., Kildare, Ireland) was used to measure Hp concentration. Albumin was determined using a Bromocresol green reagent (Albumin OSR 6102 Olympus Life and Material Science Europe GmbH Lismeehan). Finally, an automated spectrophotometric assay was used to determine PON-1 concentration (Sigma-Aldrich, St. Louis, MO, USA). All techniques had been previously validated for use in dogs [70,71,72]. All analyses were performed within 6 months since the sampling procedure.

### 2.6. Statistical Analysis

Data were analyzed through a statistical package (IBM SPSS for Windows, Ver. 25.0; Armonk, NY, USA). First, data were checked through Shapiro–Wilk and Levine tests for normal distribution and homogeneity of variances, respectively. When distribution of data was not normal and/or variances were not homogenous, they were transformed through arcsine √x or √x. Following this, a linear mixed model followed by post-hoc Sidak test was run per variable (changes in concentration of albumin and PON-1 in serum, age, and weight of animals and time of surgery). In this model, the between-subjects factor was the surgery treatment (laparoscopic ovariectomy, midline ovariectomy, and flank ovariectomy) and the within-subjects factor was the time of evaluation (before surgery, 1, 24, 72, and 168 h post-intervention). When, even after linear transformation, data did not match with parametric assumptions (changes in the concentrations of CRP and Hp in serum), Friedman and Wilcoxon tests were used as non-parametric alternatives.

Correlation between variables were calculated with Pearson coefficient when data fit with parametric assumptions (changes in concentration of albumin and PON-1 in serum, and with Spearman coefficient when distribution was not normal and/or variances were not homogeneous (changes in the concentrations of CRP and Hp in serum).

In all statistical analyses, the level of significance was set at *p* ≤ 0.05. Data are shown as mean ± standard error of the mean (SEM).

## 3. Results

**Figure 1:**
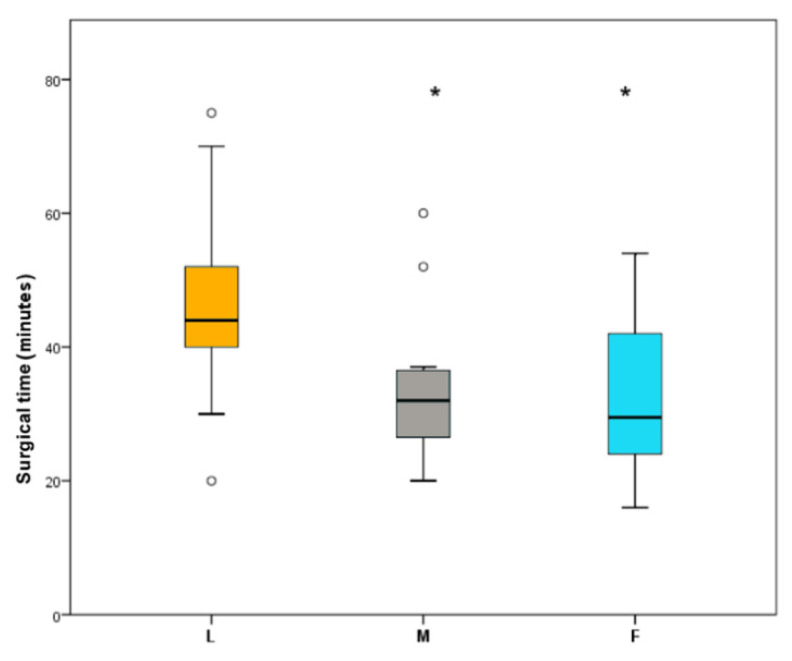
Surgical time in minutes per study group. L: laparoscopic, M: midline, F: flank. * *p* < 0.05: Statistically significant difference among groups. ° Outlier values.

Dogs aged 5 to 48 months (15.7 ± 12.1) and weighted 2.5 to 51 kg (13.7 ± 9.4). The group did not differ with regards to age (*p* = 0.58) or body weight (*p* = 1.00). The total surgical time was 26 to 52.4 min (37.1 ± 13.8). Surgical time for group L was 39.7 to 52.5 min (46 ± 3.2,); for group M was 26.8 to 40 min (33.2 ± 3.2), and for group F was 26.1 to 38.5 min (32.3 ± 3.1). Figure 1 represents the surgical time per group.

### 3.1. CRP and Hp

**Figure 2:**
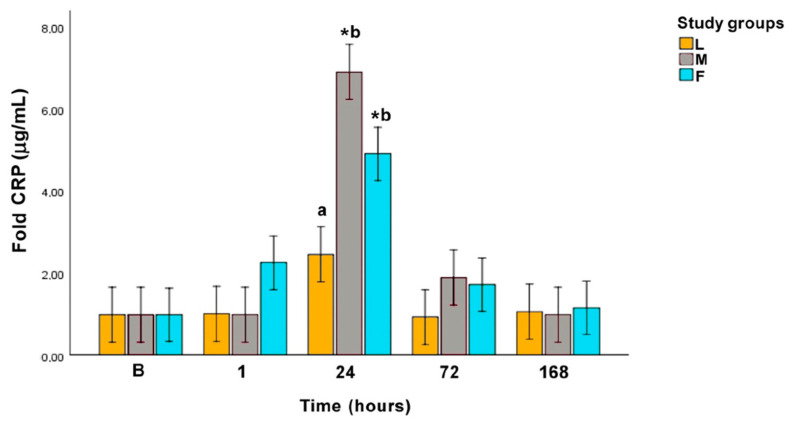
Fold C-reactive protein (CRP) concentration along the experimental period. L: laparoscopic ovariectomy (OVE), M: midline OVE, and F: flank OVE. Different letters (a, b) indicate differences between groups. B: baseline. * *p* < 0.05: statistically significant difference compared to baseline.

CRP values peaked at 24 h in all groups, although it was only statistically significant compared with baseline value in groups M (*p* = 0.001) and F (*p* = 0.001). At this time point, CRP concentration in group L was significantly lower than in groups M (*p* = 0.001) and F (*p* = 0.028). Figure 2 represents CRP in fold values.

**Figure 3:**
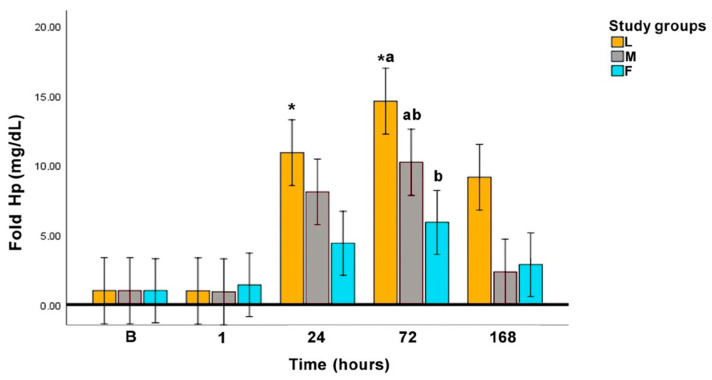
Fold Hp concentration along the experimental period. L: laparoscopic OVE, M: midline OVE, and F: flank OVE. Different letters (a, b) indicate differences between groups. B: baseline, * *p* < 0.05: statistically significant difference compared to baseline.

Hp concentration for group L was significantly increased above baseline at 24 h (*p* = 0.034) and 72 h (*p* = 0.001), showing the highest levels 72 h after the surgery. In groups M and F, Hp concentrations did not increase significantly compared to basal values. When surgical procedures were compared, statistically significant differences between L and F groups at 72 h were observed (*p =* 0.026), with L group showing higher levels of Hp than F group. Figure 3 represents Hp in fold values.

### 3.2. Alb and PON-1

**Figure 4:**
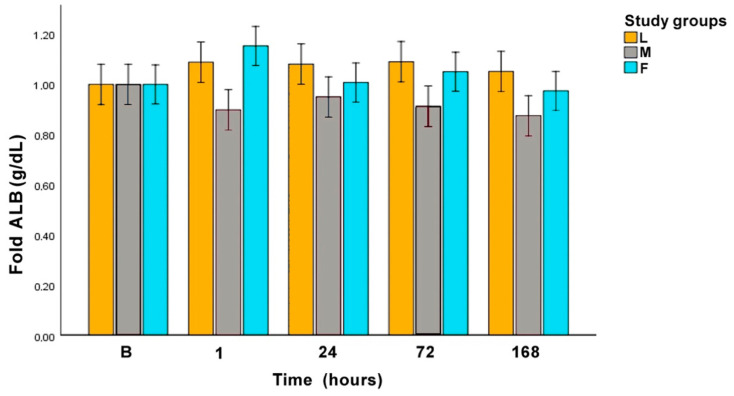
Fold albumin (Alb) concentration along the experimental period. L: laparoscopic OVE, M: midline OVE, and F: flank OVE. B: baseline.

**Figure 5:**
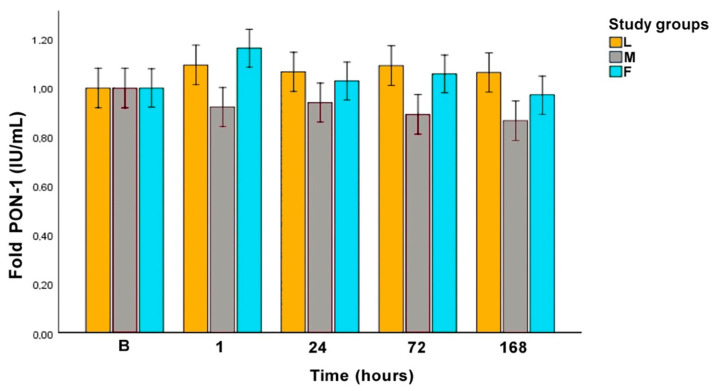
Fold paraoxonase-1 (PON-1) concentration along the experimental period. L: laparoscopic OVE, M: midline OVE, and F: flank OVE. B: baseline.

Albumin and PON-1 showed no significant differences among the sampling times in each surgical procedure, nor among the surgical procedures. Figure 4 represents Alb in fold values and Figure 5 represents PON-1 in fold values.

## 4. Discussion

Surgical neutering is a widely used method of contraception in veterinary medicine, and many bitches undergo major abdominal surgery for elective OVE yearly around the world. The traumatic surgical injury results in the release of pro-inflammatory cytokines by local monocytes and macrophages and stimulates acute inflammation and synthesis of APP [7]. According to the authors’ knowledge, this is the first study in evaluating the APR in bitches after OVE by comparing three different surgical approaches, laparotomy, midline and flank. Studying the APR after OVE using these three different techniques objectively evaluates the degree of the surgical trauma and permits to compare the post-operative inflammatory response.

CRP peaked 24 h after surgery in groups M and F, showing that a moderate inflammatory response occurs after these two open techniques, which is in agreement with that previously observed in bitches after OVH [58,61,73]. In contrast, CRP levels in group L did not undergo significant changes over the post-operative period. The present data coincides with a study confirming that CRP allows to differentiate degrees of inflammation in the post-operative period of three different soft-tissue surgical procedures. Even though the main differences between groups for that study were at 12 h post-intervention [10]. In human medicine, a smaller elevation in CRP was detected after laparoscopic hysterectomy compared with the open abdominal procedure [74]. In veterinary medicine, several different laparoscopic procedures have showed lower concentrations of CRP compared with open surgical techniques [13,15,60], which is in agreement with the present results.

As it has been previously stated, Hp can increase from 24 h on after surgery, reaching the peak 3–4 days later. [5] In accordance with this, peak level for Hp in the present study was reached 72 h (3 days) post-intervention. At this time point, Hp levels in group L were higher than in groups M and F. Hp strongly blinds free hemoglobin resulting in the complex HP-hemoglobin [15,75,76]. Thus, free Hp in serum may decrease on situations of hemorrhage or hemolysis, making that determination by hemoglobin binding assays may be unreliable [3,5,77,78,79]. In M and F groups, a higher degree of hemolysis immediately after surgery would be expected due to larger tissue injury, whereas in the L-group, the better viewing of the abdominal cavity and vessels implies less manipulation of the tissues. As previous studies have reported, this would imply that a hemorrhage is less probable [80]. Surgical hemorrhage or intraoperative blood loss can be quantified using colorimetric and gravimetric methods. However, the blood loss was not measured in none of the surgeries, being a limitation of the study [81].

Another fact that must be elicited is that bitches in group L were subjected to the effect of the pneumoperitoneum. The insufflation of CO_2_ into the abdominal cavity has numerous effects both local and systemic, mainly an ischemia-reperfusion injury which is caused by decreased splenic blood flow and organ ischemia due to oxidative stress [82,83]. Visceral pain and discomfort are reported in human medicine after laparoscopic procedures [84] and it has been proved that the morphologic integrity of the peritoneum is transiently altered after CO_2_ pneumoperitoneum in mice [85]. This phenomenon is less studied in veterinary medicine, but could explain the results of Hp obtained in group L. A study in rats subjected to pneumoperitoneum showed an increase of inflammatory cytokines and histopathological changes in lung tissue, with an oxidative status that correlated with the degree of intra-abdominal pressure [53]. Further studies on the effect of the pneumoperitoneum in dogs are needed, so its role in post-operative pain could be better understood.

Alb levels did not change among the sampling times neither among surgical procedures. This APP is mainly affected by chronic status rather than acute damage [86], so maybe the possible variations had not occurred yet. On the other hand, being a marker for chronic damage would explain the scarce literature regarding Alb after surgical trauma in dogs. In addition, none of the dogs included in the study reported surgical post-operative complications, being another possible reason for Alb levels to remain stable over the complete study period.

PON-1 did not undergo significant changes during the post-operative period in none of the studied groups. This is in accordance with a study where PON-1 levels, 4 days after surgery, were not different from basal values [23]. The lack of variations could be explained by the fact that OVE procedure does not produce enough oxidative stimulus. Even though, a trend for lower levels of PON-1 were seen in group M and F compared to group L at 24, 72, and 168 h.

It has been seen that surgical inflammatory response is affected by surgical time, thus, the differences in surgical time may have an impact in the results [57]. There is no agreement in terms of the duration of laparoscopic and laparotomic OVE. Some studies report shorter surgical times in laparoscopic compared to laparotomic procedures, but the latter were done using ligatures instead of sealing devices for the pedicles and that could bias the result [39]. Other studies report longer surgical times in laparoscopic OVE likely due to the learning curve associated to this technique [37]. In this study, laparoscopic OVE took longer than midline and flank OVE. However, CRP levels were lower in the post-operative time for L group. No correlation between surgical time and APP has already been described in previous studies [42,87,88]. The use of the LigaSure™ (Medtronic, [formerly Covidien], Minneapolis, MN, USA) sealing device in the open OVE groups could explain the shorter time described, as these conventional procedures are usually performed using ligatures rather than vessel sealing devices. The surgeon’s experience is also important as it is intimate related to the length of the surgery and to the trauma caused to the tissues [89]. In this project, the experience of the surgeon, the small size of the wound and the use of the vessel sealing device in the open technique, could justify the results regarding surgical time.

Reference intervals have been established for some species [90,91], and also some interbreed variation of APP and other biomarkers appear in the literature [92,93]. There were 15 different breeds represented in the study and no statistical difference related to breed has been observed. In addition, to avoid a possible breed or individual effect, values were corrected by basal value for each APP (fold values). Likewise, no significant differences have been described in APP in terms of age and sex [94].

Minimizing tissue trauma and successive stress response is an important goal of surgery [89]. Currently, many surgical procedures in veterinary medicine are performed by laparoscopic procedures, and laparoscopic OVE is commonly preferred by pet owners when deciding to neuter their pet [47,68]. The results from the present study show that open OVE by midline and flank approaches produce a moderate and similar post-surgical inflammation based on the CRP values measured, being these values higher than those observed after laparoscopic OVE.

## 5. Conclusions

On the basis of the findings in this study, the authors conclude that concentrations of CRP in serum of bitches when performing laparoscopic OVE are lower than when midline and flank approaches are used. However, Hp concentrations may need further studies to understand the possible influence of blood loss on this parameter. Thus, based on the results of CRP changes, laparoscopy would be a favorable technique over conventional open approaches for OVE in the bitch.

## Figures and Tables

**Table 1:** Descriptive data of breed for each patient.

Dog	Breed	Dog	Breed	Dog	Breed
1	Crossbreed	17	Shih-Tzu	32	Podenco
2	Crossbreed	18	Mastiff	33	Crossbreed
3	Crossbreed	19	Maltese	34	Crossbreed
4	Crossbreed	20	Crossbreed	35	Yorkshire terrier
5	Weimaraner	21	Podenco	36	Crossbreed
6	Am Staffordshire	22	Am Staffordshire	37	Siberian husky
7	Podenco	23	Beagle	38	Podenco
8	Bull Terrier	24	Yorkshire terrier	39	Crossbreed
9	Crossbreed	25	Jack Russell	40	Crossbreed
10	Spaniel Breton	26	Yorkshire terrier	41	Crossbreed
11	Crossbreed	27	Crossbreed	42	Podenco
12	Crossbreed	28	Crossbreed	43	Podenco
13	Am. Staffordshire	29	Podenco	44	Mastiff
14	Pinscher	30	Podenco	45	Crossbreed
15	Crossbreed	31	Greyhound	46	Crossbreed
16	Crossbreed	32	Dachshund		
